# Intraosseous lipoma of the third lumbar spine: a case report

**DOI:** 10.1186/s13256-015-0528-5

**Published:** 2015-03-06

**Authors:** Chaipond Teekhasaenee, Koji Kita, Kenji Takegami, Eiji Kawakita, Toshihiko Sakakibara, Yuichi Kasai

**Affiliations:** 1 Department of Spinal Surgery and Medical Engineering, Mie University Graduate School of Medicine, 2-174 Edobashi, Tsu city, Mie 514-8507 Japan; 2 Department of Orthopedic Surgery, Thammasat University Hospital, 2 Prachan Road, Bangkok, 10200 Thailand; 3 Department of Orthopedic Surgery, Saiseikai Matsusaka General Hospital, 1-15-6 Asahi-cho, Matsusaka city, Mie 515-8557 Japan

**Keywords:** Intraosseous lipoma, Lumbar spine, Benign spinal tumor, Surgery, Low back pain

## Abstract

**Introduction:**

Intraosseous lipoma is a benign bone tumor, and the tumor occurs more frequently in the lower extremities. We present a very rare case of intraosseous lipoma occurring in the lumbar vertebral arch and spinous process.

**Case presentation:**

A 54-year-old Japanese man presented with a three-month history of lumbar pain. Magnetic resonance imaging of the L3 vertebral arch and spinous process revealed high intensity on T1- and T2-weighted imaging, and it was suppressed on fat-suppression imaging and no enhancement showed on gadolinium contrast-enhanced imaging. Computed tomography imaging revealed an osteolytic change accompanied by marginal osteosclerosis in his third lumbar vertebral arch and spinous process, as well as a thinned and bulging bone cortex. An analgesic had been administered prior to his visit, but low back pain had persisted, so we performed curettage and filled the defect with hydroxyapatite bone. His low back pain was improved immediately after surgery, and no recurrence of tumor has been observed on computed tomography imaging as of three years postoperatively.

**Conclusions:**

Symptomatic intraosseous lipoma of spine is very rare, but the patient may be surgically well-treated by curettage and reconstruction of the benign tumor.

## Introduction

Intraosseous lipoma is a benign bone tumor, and the tumor occurs more frequently in the lower extremities. We present a very rare case of intraosseous lipoma occurring in the lumbar vertebral arch and spinous process, together with a discussion of the literature.

## Case presentation

A 54-year-old Japanese man presented to our university-affiliated hospital with a three-month history of lumbar pain. He was 167cm in height and weighed 58kg. An analgesic had been administered prior to his visit, but low back pain had persisted. His physical examination showed pressure pain and tapping tenderness at the third lumbar vertebral level, but no sensory or motor disorders of his lower extremities. His blood biochemistry showed no abnormalities and his medical history was non-contributory.

**Figure 1:**
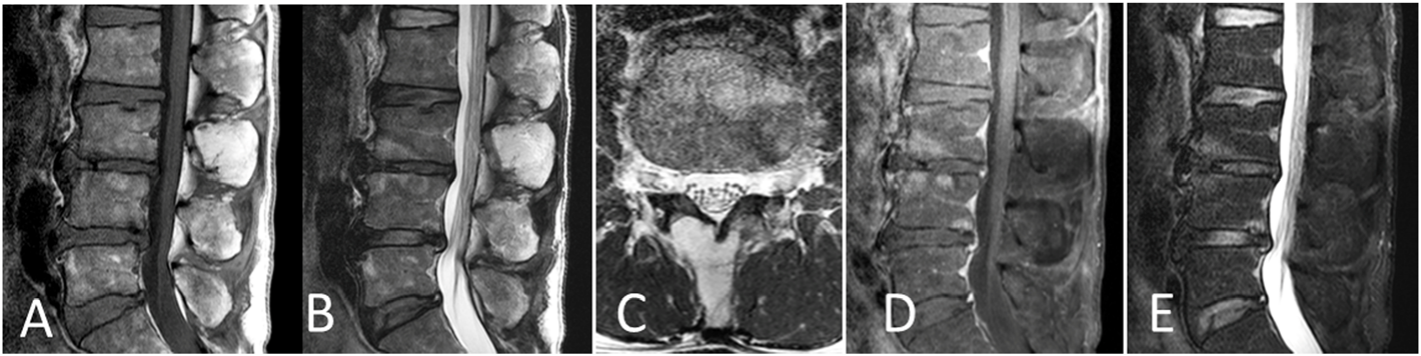
**Magnetic resonance imaging at the L3 vertebral arch and spinous process. (A)** Sagittal T1-weighted image; **(B)** Sagittal T2-weighted image; **(C)** Axial T2-weighted image; **(D)** Sagittal fat suppression image; **(E)** Sagittal T1-weighted gadolinium contrast (+) image.

**Figure 2:**
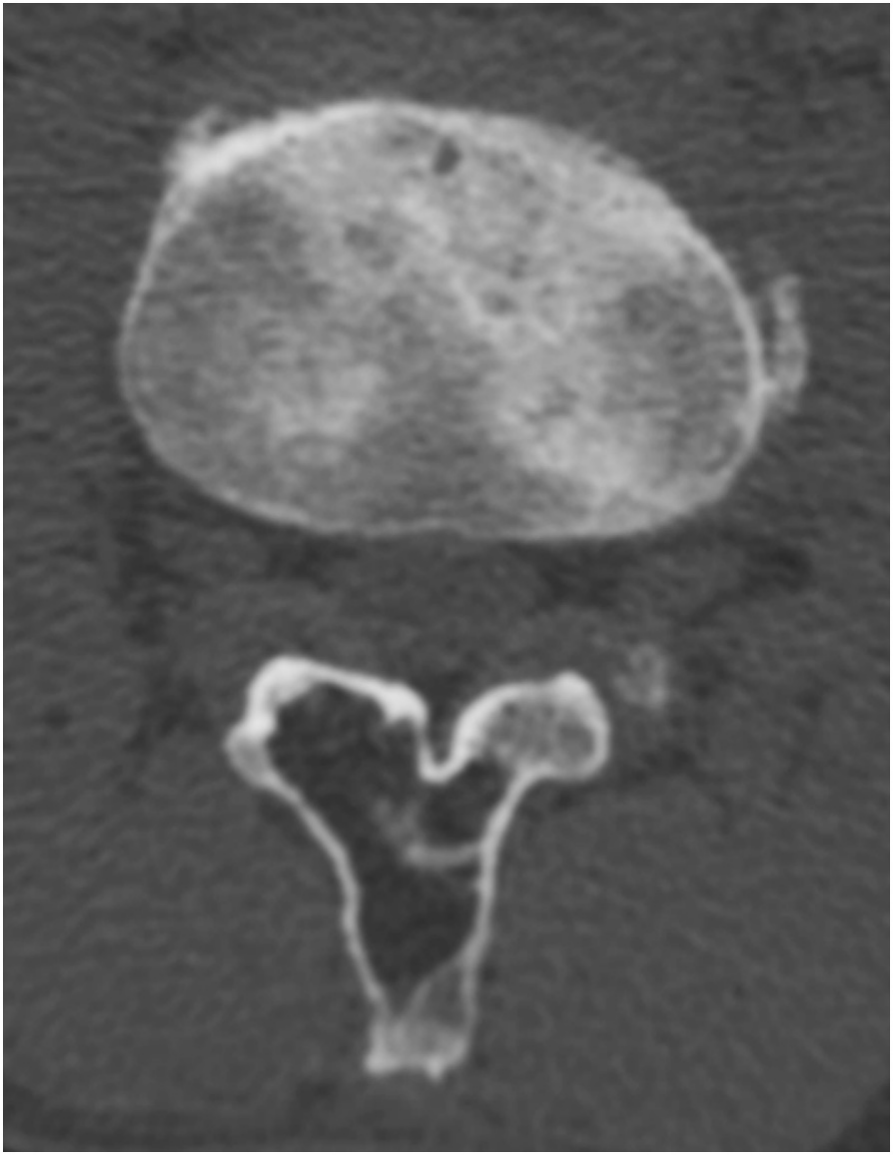
**Computed tomography image revealing an osteolytic change accompanied by marginal osteosclerosis in his third lumbar vertebral arch and spinous process, as well as a thinned and bulging bone cortex.**

A plain radiography revealed the formation of a vertebral spur or narrowing of the intervertebral disc between L3 and L4 as an age-related change, but no instability was evident between vertebrae and no obvious abnormalities were evident. Magnetic resonance imaging (MRI) of the L3 vertebral arch and spinous process revealed high intensity on T1- and T2-weighted imaging (Figure 1A,B,C), and it was suppressed on fat-suppression imaging (Figure 1D) and no enhancement showed on gadolinium (Gd) contrast-enhanced imaging (Figure 1E). Computed tomography (CT) imaging revealed an osteolytic change accompanied by marginal osteosclerosis in his third lumbar vertebral arch and spinous process, as well as a thinned and bulging bone cortex (Figure 2). Hounsfield units (HU) of CT for the area at which the osteolytic change was observed was −87HU, a value approximating that of fatty tissue, and areas of ossification or calcification were observed.

Based on the above findings, although we suspected painful lipoma in the third lumbar vertebral arch and spinous process segment, we decided to perform a biopsy to confirm the diagnosis. Since a benign tumor was suspected, we planned to perform curettage of the tumor and to fill the defect with artificial bone.

**Figure 3:**
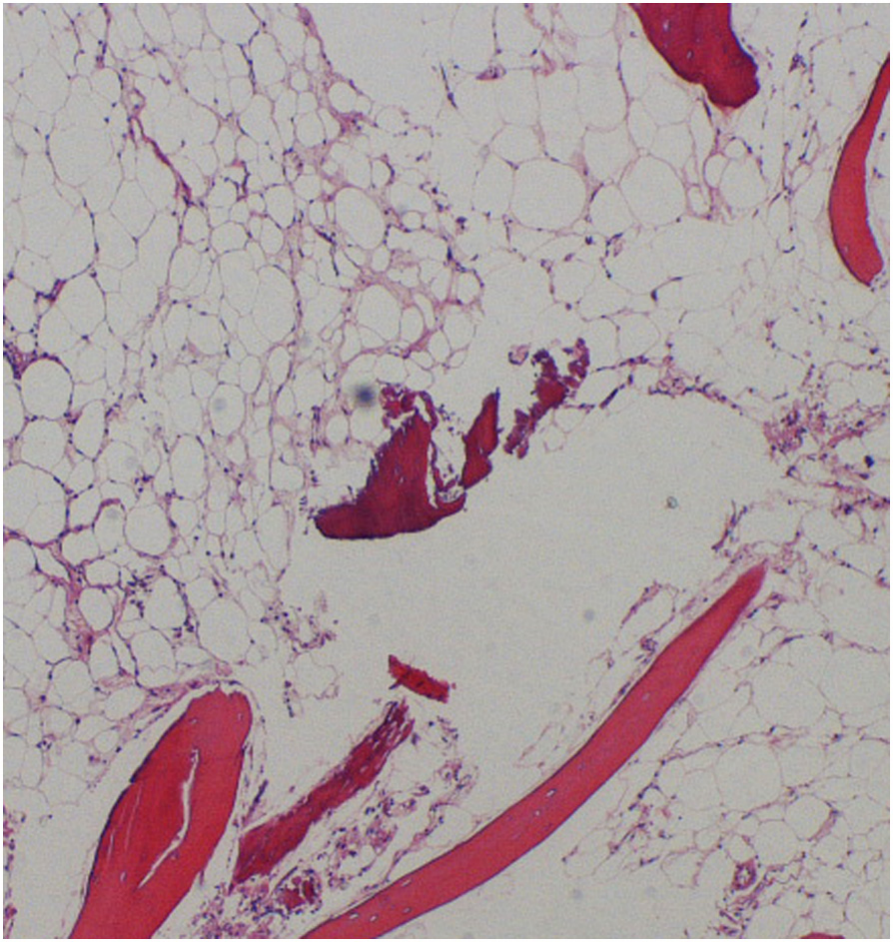
**Intraoperative pathological findings of hyperplasia of adipose cells and blood vessels, a small amount of trabecular bone and adipose cells of different sizes.** (Hematoxylin and eosin ×4 magnification).

The operation was performed under general anesthesia. The third lumbar vertebral arch was exposed, and when an area approximately 1cm × 1cm in the external lamina of the right vertebral arch was opened, a yellow tumorous lesion with a color and elasticity macroscopically similar to those of ordinary fatty tissue was observed. The tumorous lesion was curetted away as much as possible, hydroxyapatite bone filler paste (BIOPEX®; HOYA Corporation, Tokyo, Japan) was used to fill the defect and the external lamina of the vertebral arch was replaced. Intraoperative pathological findings included hyperplasia of adipose cells and blood vessels, a small amount of trabecular bone and adipose cells of different sizes. Intraosseous lipoma was therefore diagnosed (Figure 3).

**Figure 4:**
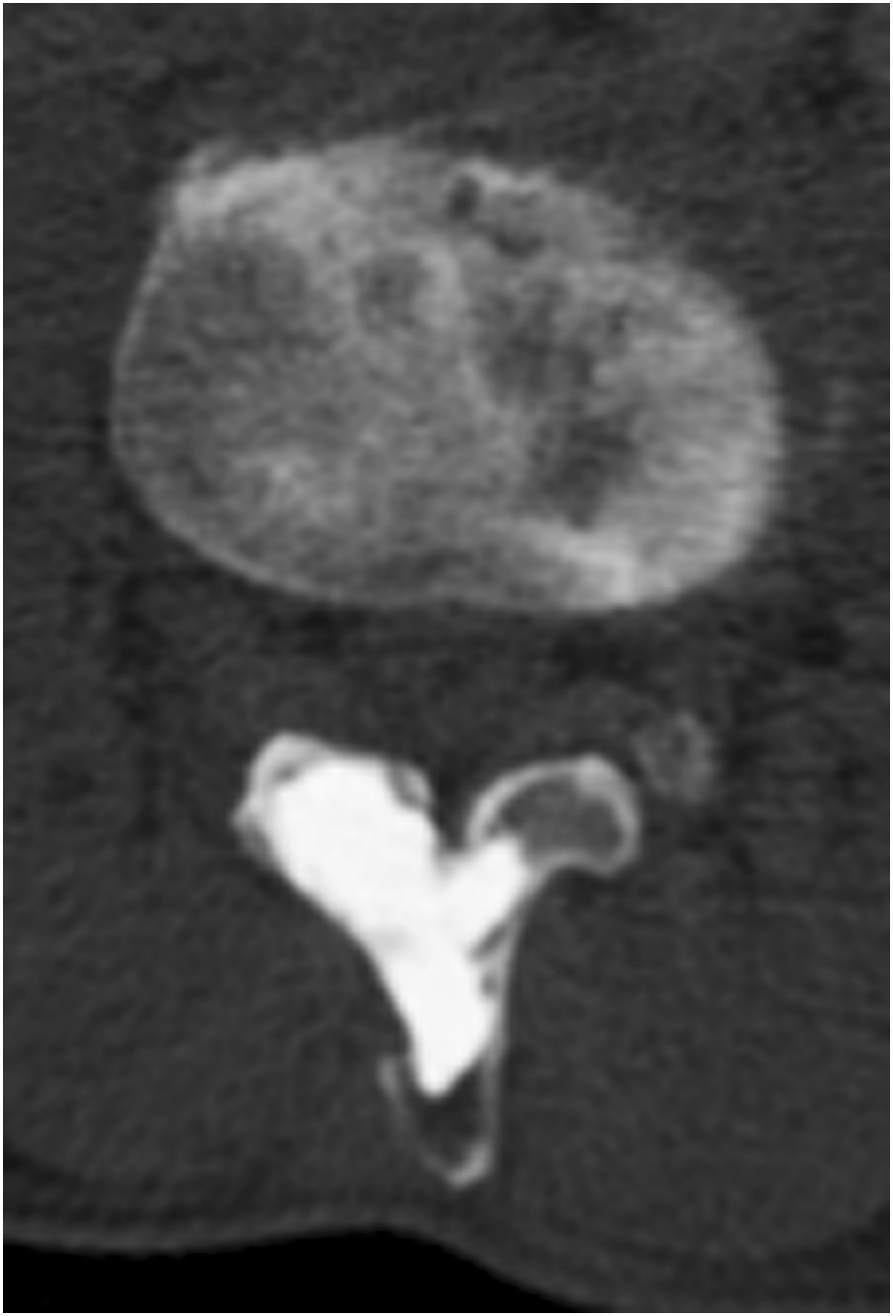
**Computed tomography image three years after surgery.**

His low back pain was improved immediately after surgery, and no recurrence of the tumor has been observed on CT imaging as of three years postoperatively (Figure 4).

## Discussion

Despite the abundance of adipose connective tissue in bone marrow, intraosseous lipoma is extremely rare [1]; a search of PubMed using the keywords ‘intraosseous lipoma’ yielded 177 results. A review of the search results showed that intraosseous lipoma occurs more frequently in the lower extremities, particularly in the calcaneus [2] and metaphysis of long bones [3]. Campbell *et al*. [2] reported that lipomas occur most frequently in the calcaneus (32%), while Milgram [3] found that lipomas occur most frequently in the metaphysis of the proximal femur (34%).

We performed a review of the literature on intraosseous lipoma involving the spine, identifying only 14 cases (Table 1); five cases (35%) occurred in the lumbar region, four (28%) in the sacral region, three (21%) in the cervical region, one (7%) in the thoracic region and one (7%) in the coccygeal region [4-14]. A slight predominance towards the lumbar spine was seen compared with other regions. The lesion in our patient also occurred in the lumbar region. Intraosseous lipoma in the lumbar region might occur at the vertebral body or in the posterior element [8,12], with a slight predominance toward the vertebral body. However, our patient presented with the lesion in the posterior element.

**Table 1:** **Spinal intraosseous lipoma reported in the literature**

**Author**	**Published year**	**Patient’s age**	**Gender**	**Site of involvement**	**Treatment**
Bin *et al*. [4]	2010	27	Male	C1-2 vertebral body	Curettage and reconstruction
Lin *et al*. [5]	2009	37	Female	C3 spinous process	Surgery
Chang and Park [6]	2003	38	Male	T1 lamina	Excision
Kamekura *et al*. [7]	2002	49	Male	Sacrum	Excision
Pande *et al*. [8]	1998	35	Male	L1-2 vertebral body	Biopsy
Williams *et al*. [9]	1993	45	Male	L1 vertebral body and neural arch	Biopsy
Williams *et al*. [9]	1993	38	Female	L4 vertebral body	Observation
Williams *et al*. [9]	1993	47	Male	L4 vertebral body	Observation
Milgram [10]	1991	28	Female	Sacrum	Biopsy
Ehara *et al*. [11]	1990	53	Male	Sacrum	Biopsy
Milgram [3]	1988	20	Male	C2 vertebral body	N/A
Matsubayashi *et al*. [12]	1980	27	Male	L4 spinous process	Laminectomy
Hanelin *et al*. [13]	1975	33	Male	Coccyx	Coccygectomy
Zorn *et al*. [14]	1971	21	Male	Sacrum	Biopsy

Even though intraosseous lipoma is a benign tumor that can be successfully treated with conservative treatment, surgery has been recommended for diagnostic confirmation, painful tumors, pathological fractures and malignant transformation [1-3,15,16]. In our patient, low back pain persisted after conservative treatment, so we performed curettage and filled the defect with hydroxyapatite bone. Most cases of intraosseous lipoma have no pain, however, micro-movement of the periosteum of the L3 vertebral arch and spinous process may have caused our patient’s pain. Subsequently, the low back pain of our patient was improved immediately after filling the curetted defect with hydroxyapatite, and no recurrence of tumor has been observed as of three years postoperatively.

## Conclusions

Symptomatic intraosseous lipoma of spine is very rare, but the patient may be surgically well-treated by curettage and reconstruction of the benign tumor.

## Consent

Written informed consent was obtained from the patient for publication of this case and accompanying images. A copy of the written consent is available for review by the Editor-in-Chief of this journal.

## Abbreviations

CT: Computed tomography
HU: Hounsfield units
MRI: Magnetic resonance imaging
